# Utilization of mental health services and associated factors among residents of southern Ethiopia; a community based cross-sectional study

**DOI:** 10.1186/s12913-025-12400-w

**Published:** 2025-02-15

**Authors:** Birhanu Alamirew, Beniam D. Darge, Bezabih Terefe, Feleke Gebremeskel

**Affiliations:** 1 School of Nursing, College of Medicine and Health Sciences, Arba Minch University, Arba Minch, Ethiopia; 2 Global Brain Health Institute, University of California San Francisco, San Francisco, CA USA; 3 Department of Midwifery, College of Medicine and Health Sciences, Arba Minch University, Arba Minch, Ethiopia; 4 School of Public Health, College of Medicine and Health Sciences, Arba Minch University, Arba Minch, Ethiopia

**Keywords:** Treatment gap, Mental health, Depression, Anxiety, Ethiopia

## Abstract

**Background:**

In 2019, nearly one billion individuals worldwide were estimated to be living with some form of mental illness. This staggering figure underscores not only the widespread prevalence of mental health issues but also their significant negative impact. Despite the critical impact of mental health problems, there is a substantial gap in treatment at the global scale. Particularly in rural Ethiopia, there is a notable lack of data regarding the use of mental health services by community residents. This study was conducted with the aim of evaluating the utilization of mental health services and identifying factors that influence access to these services among the population of rural southern Ethiopia.

**Methods:**

A community-based cross-sectional study was conducted on randomly selected adults in southern Ethiopia. A semi-structured questionnaire assessing the sociodemographic status of the participants was used. Depression and anxiety were measured with the Patient Health Questionnaire (PHQ-9) and the General Anxiety Disorders Scale (GAD-7), respectively. The data were then analyzed using StataMP version 18. Statistical tests such as frequency, percentage, mean, bivariate logistic regression, and multivariate analysis were performed. P values and confidence intervals were used to determine statistical significance.

**Results:**

A total of 971 participants were enrolled in this study, and the mean age was 34.9 (± 11) years. A minority of the participants (152 [15.5%]) sought some form of help for mental health-related reasons. Only 24 (2.5%) of the participants used biomedical mental health services. The perceived need for any mental health service was 96 (9.9%). Thirty-three percent of the respondents with mild to moderate depression and 37.1% of those with severe depression sought care for their concerns. Similarly, 37% and 35% of individuals with mild to moderate anxiety and severe anxiety, respectively, utilized mental health services. A higher monthly income, psychoactive substance use, perceived need, and symptoms of depression and anxiety were significantly associated with mental health service utilization.

**Conclusion:**

Our study revealed a significantly low level of utilization of mental health services among the residents of rural southern Ethiopia. More efforts to address the treatment gap for mental health problems in the country are needed.

**Supplementary Information:**

The online version contains supplementary material available at 10.1186/s12913-025-12400-w.

## Background

In 2019, it was estimated that nearly one billion people lived with some form of mental illness globally [1, 2]. This number has increased by more than 25% during and after the COVID-19 pandemic, with a similar increasing trend in the prevalence of mental illness expected to continue [3–5].

Approximately 75% of adults with a mental health disorder experience the onset of the problem before 24 years of age, indicating that most mental health problems start in childhood and adolescence [6, 7]. According to a recent report in the United States, in 2022, approximately 4.8 million adolescents were experiencing symptoms of depression, and approximately 48.7 million people older than 12 years were living with substance use disorders [8].

The prevalence and magnitude of mental illness are also increasing in Low- and Middle-Income Countries (LMICs), including sub-Saharan Africa [1, 9]. Similarly, recent studies have shown that a significant proportion of the population in Ethiopia is affected by mental illness [10–12]. One study in southern Ethiopia reported that the prevalence of common mental disorders was 39.7% [13].

In addition to their widespread prevalence, mental health issues and substance abuse significantly impair the quality of life for both patients and their caregivers [14, 15]. Recent data suggest that mental illnesses account for 418 million disability-adjusted life years (DALYs), representing 16% of the global total. The financial implications are equally staggering, with the cost of mental health conditions reaching an estimated USD 5 trillion in 2019. This expenditure erodes a considerable portion of the national GDP, diverting resources that could have been allocated to other critical areas [16].

Despite the increasing global burden of mental illnesses, these conditions have not received the same level of attention as physical diseases and have been largely overlooked, particularly in (LMICs) [2, 17, 18]. There is a pronounced disparity worldwide between the number of people requiring mental health care and those who access treatment services [8, 18–20]. This treatment gap highlights a critical issue in the global health landscape, underscoring the need for enhanced recognition and resources dedicated to addressing mental health.

A recent study by the World Health Organization (WHO) indicated that only 36.8% of the population in high-income countries and 22% of those in upper-middle-income countries utilized mental health care [19]. Similar studies from high-income countries, including the United States, Canada, Australia, Switzerland, and the Czech Republic, also reported a significant gap in the treatment of mental health problems [6, 20–23].

Help-seeking behavior and the utilization of mental health care services are notably low, particularly in LMICs. According to a WHO mental health survey, the utilization rate of mental health services was estimated at a mere 13% [19]. Other studies have echoed these findings, consistently indicating that the actual use of mental health services falls significantly below expectations [17, 24, 25].

In the related literature, several factors have been shown to contribute to individuals’ utilization of mental health care services. These factors can be divided into several groups, including individual factors such as age and education and environmental, cultural, social, and economic factors. In addition, systemic and structural factors are implicated, including the availability and affordability of services. In addition, studies have indicated that awareness of the causation and treatment of the condition, as well as the stigma associated with mental illnesses, are contributing factors [6, 17, 18, 26].

Although efforts have been made to address some of these challenges regarding individuals’ access to and utilization of these services, the problem persists. These efforts have involved integrating mental health care services into primary health care, implemented in both developed and low-resource settings, and there is evidence for their effectiveness [18, 27, 28]. However, there is still a large treatment gap.

In Ethiopia, the extent of these challenges has been the subject of various studies revealing levels of care utilization. For example, one study reported that a mere 4.2% of mothers experiencing postpartum depression sought help, whereas another indicated that only 13% of individuals with an alcohol use disorder sought treatment [29, 30]. A qualitative investigation in northwestern Ethiopia highlighted the generally low propensity for seeking mental health care, identifying several reasons behind this reluctance [26]. Despite these insights, data on the broader utilization of mental health treatment services among the general population are scarce. This gap prompted the current study, which aims to explore the use of mental health services among community residents in southern Ethiopia.

## Method

### Aim, design, and setting

This study aimed to assess the utilization of mental health services and identify the factors associated with these services among residents of rural southern Ethiopia. It was conducted as a community-based cross-sectional study in two districts in the Gamo Administrative Zone. The districts are part of an existing Arba Minch Health and Demographic Surveillance System (AM-HDSS). The districts are 405 km south of the national capital, Addis Ababa, and have mixed highland, midland, and lowland areas. The data were collected between October 2021 and May 2022.

### Participants

All residents of the two districts who were at least 18 years of age and resided in the area for at least six months before the data collection were eligible to participate in the study. The only exclusion criterion was a critical medical illness that prevented participation and providing a response. A single population proportion formula was used to calculate the optimal sample size for the study. The following assumptions were used: a prevalence rate (P) of 33.6% for common mental disorders in southwest Ethiopia [31], a reliability level of 99%, a design effect of 1.5, and the addition of a 10% nonresponse. As a result, the calculated sample size for this study was 976.

Multistage systematic random sampling was employed to select the study participants. The AM-HDSS has nine Kebeles (the lowest administrative units in the government structure) in the two districts. First, five out of the total nine kebeles were selected using the lottery method. Next, the calculated sample was proportionally allocated to the five selected kebeles according to their total number of households. A systematic sampling technique was used to select households to be included by calculating a k value. Finally, only one participant was recruited into the study from a household using the lottery method in households with more than one eligible individual.

### Process

Ten data collectors and five trained field supervisors collected the data using the Open Data Kit (ODK) software on a smartphone by going from house to house. Written informed consent was obtained before data collection, and participation was voluntary. The desired information from the selected sample participants was collected through face-to-face interviews using a semi-structured questionnaire for sociodemographic data, which consisted of questions about age, sex, marital status, occupation, educational level, income, etc.

Mental health service utilization was estimated using a modified version of the mental health services and psychotropic medication components of the Canadian community health survey questionnaire [32]*.* The modified questionnaire included a “yes” or “no” question, which was phrased as follows: “Have you ever received care for mental health-related reasons from a psychologist, doctor, friend, family, religious leader, traditional healer, or other person?”. For this purpose, mental health service utilization was defined as receiving any form of mental health service, such as counseling or medication, from a professional mental health provider, such as a psychiatrist or trained mental health provider, and/or receiving help from friends, family, religious leaders, traditional healers and/or others.

To determine need factors, participants were asked about depression and anxiety symptoms as well as their perceived need for mental health services. The Patient Health Questionnaire (PHQ-9) is a nine-item depression screening questionnaire with higher scores indicating higher depression symptoms. The seven-item General Anxiety Disorder (GAD-7) tool was used to screen for symptoms of Anxiety [33, 34]. Perceived need was defined as how individuals perceive their mental health and the importance of receiving treatment. The answer was determined with a yes/no question: “Was there ever any time when you thought you needed mental health treatment or counseling of any kind?”.

### Analysis

The data were imported from the database, cleaned, and analyzed using Stata M.P. version 18 statistical software. Descriptive statistics such as frequency and percentage were computed, and bivariate logistic regression analysis was conducted for each independent variable and outcome of interest (mental health service utilization). A *p*-value < 0.25, context, and previous studies were considered to indicate which variables should be included in the final multivariable logistic regression model. In the final model, a *p*-value < 0.05 and confidence intervals were used to indicate statistical significance. The crude and adjusted odds ratios with 95% confidence intervals were computed and interpreted accordingly. The goodness of fit was measured by the Hosmer–Lemeshow test.

## Results

A total of 971 individuals with a 99.5% response rate participated in the study. The mean age of the respondents was 34.9 (± 11) years, with a minimum of 18 years and a maximum of 77 years. Among the total participants, 576 (59.3.6%) were male, and most respondents were married (66 [78.9%]). Only 82 (8.4%) of the participants in our study had completed secondary school. The complete sociodemographic characteristics of the study participants are provided in the table below (Table 1).

**Table 1:** Sociodemographic characteristics of respondents residing in AMU-HDSS, Southern Ethiopia, 2022 (*N* = 971)

Characteristics	Category	Frequency	Percent (%)
Sex	Male	576	59.3
	Female	395	40.7
Age	18–39	691	71.2
	40–59	240	24.7
	60 and above	40	4.1
Marital status	Single	158	16.3
	Divorced	20	2.1
	Married	766	78.9
	Widowed	27	2.8
Religion	Orthodox	280	28.8
	Protestant	669	68.9
	Other	22	2.3
Educational status	No formal education	276	28.4
	Primary education	613	63.1
	Secondary education and above	82	8.4
Occupational Status	Employed	621	64.0
	Unemployed	350	36.0
Monthly Income	< 2,000 Ethiopian Birr	692	71.3
	2,000 – 3,999 Ethiopian Birr	190	19.6
	≥ 4,000 Ethiopian Birr	89	9.2

### Mental health service need and utilization

Among the total participants, 303 (31.2%) had a score ≥ 5 on the PHQ-9 for depressive symptoms, and 250 (25.7%) had a score ≥ 5 on the GAD-7 for symptoms of anxiety. More than one-fourth (29.7%) of the participants used one or more psychoactive substances during their lifetime.

Overall, the results of our study revealed that 152 (15.5%) of the participants sought some form of help for mental health-related concerns. Among these, a significant majority, 128 (84.2%), of the participants sought nonhealthcare mental health services, such as religious leaders and/or traditional healers, friends/family, and others. The remaining 24 (15.8%) of the respondents who received care received biomedical treatment from healthcare professionals. Biomedical treatment was defined as any form of treatment received from a trained health care professional in the form of counseling, medication, other forms of non-pharmacological care, or any combination of those. Only four of them received services from trained mental health professionals.

When analyzing the service utilization pattern, 90/268 (33.6%) of the respondents who had mild-moderate depressive symptoms sought care for their concerns, among whom only 18 (20%) went to a health facility, and the rest sought care from nonmedical sources. Similarly, 13 (37.1%) of the respondents with severe depressive symptoms received mental health care, of whom only four accessed modern mental health care services (Table 2).

**Table 2:** Mental health service utilization among respondents in AMU-HDSS, southern Ethiopia, 2022 (*n* = 971)

Variable		Sought care for mental health concerns: freq (percent)					Chi^2^ (*P*-value)
		Yes			No	Total	
		Biomedical	Non-biomedical	Total			
Depression	No depression	2 (0.2)	47 (4.8)	49 (5.1)	619 (63.7)	668(68.8)	
	Mild-moderate depression	18 (1.8)	72 (7.4)	90 (9.3)	178 (18.3)	268 (27.6)	
	Severe depression	4 (0.4)	9 (0.9)	13 (1.3)	22 (2.3)	35 (3.6)	
	Total	24 (2.5)	128 (13.2)	152 (15.7)	819 (84.3)	971 (100)	112.5 (< 0.001)
Anxiety	No Anxiety	7 (0.7)	51 (5.3)	58 (6)	663 (68.3)	721 (74.3)	
	Mild to moderate anxiety	15 (1.5)	74 (7.6)	89 (9.1)	147 (15.2)	236 (24.3)	
	Severe anxiety	2 (0.2)	3 (0.3)	5 (0.5)	9 (0.9)	14 (1.4)	
	Total	24 (2.5)	128 (13.2)	152 (15.7)	819 (84.3)	971 (100)	122.8 (< 0.001)
Perceived need for mental health services	No	16 (1.7)	87 (8.9)	103(10.6)	772 (79.5)	875 (90.1)	
	Yes	8 (0.8)	41 (4.3)	49 (5.1)	47 (4.8)	96 (9.9)	
	Total	24 (2.5)	128 (13.2)	152 (15.7)	819 (84.3)	971 (100)	101.0 (< 0.001)

The same was true for the respondents with anxiety symptoms, in which 89/236 (37.7%) of the respondents with mild to moderate anxiety sought some form of care for their concerns, among which 15 (16.8%) were from health facilities. Close to five out of 14 respondents with severe anxiety symptoms sought care, and two of them were receiving biomedical treatments.

On the other hand, 96 (9.9%) of the study participants expressed a perceived need for mental health-related needs, and the rest expressed no need for such services. Among those with a perceived need, slightly more than half (49; 51%) sought care for their concern, among whom eight received modern health care (Table 2).

**Fig. 1:**
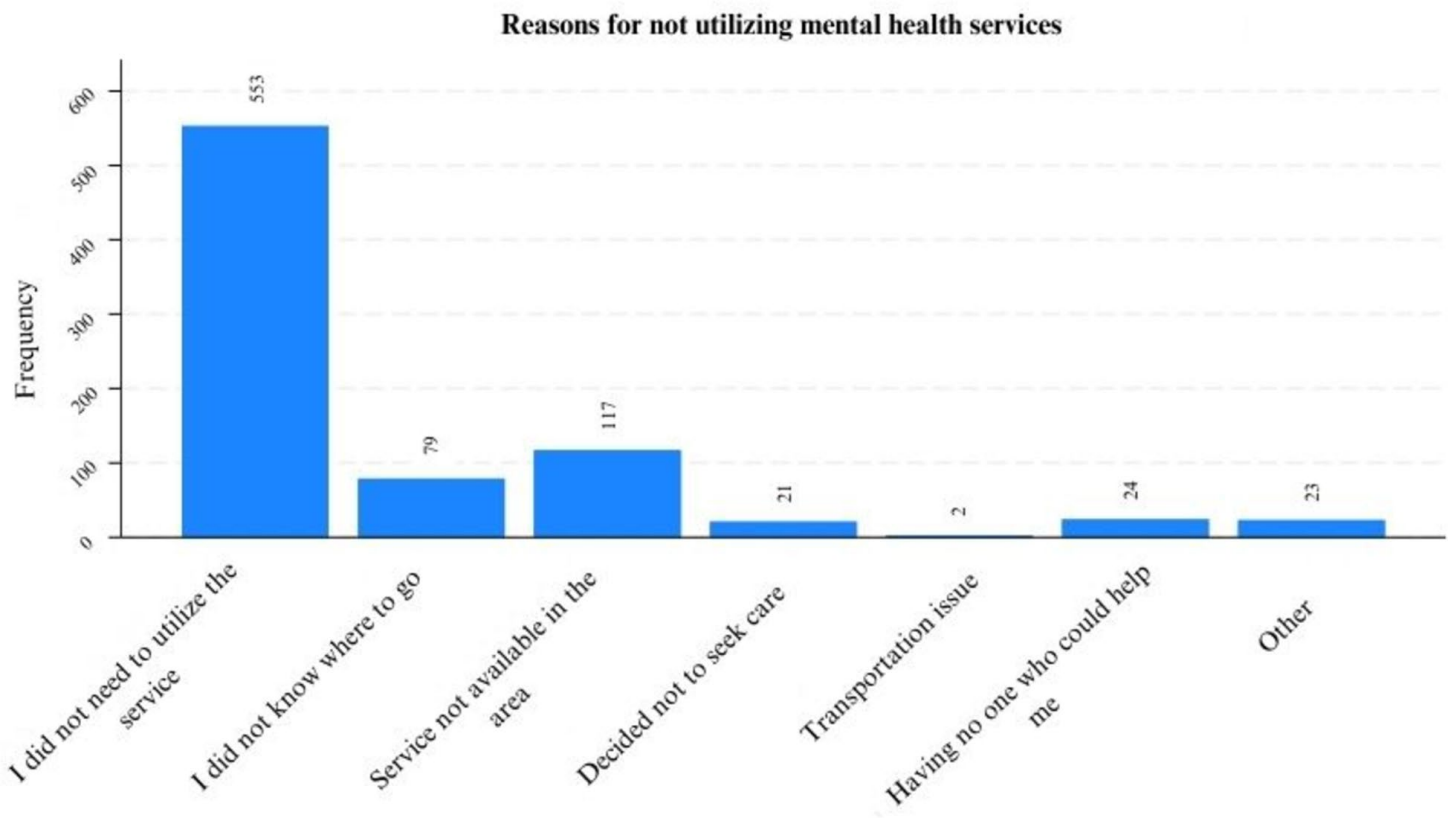
Reasons for not utilizing mental health care services among study participants in the AM-HDSS, 2022

The participants who reported never seeking mental health-related help were further asked about their reasons for not doing so. A significant majority, 553 (67.5%), reported no perceived need for services. The other common reasons were not knowing where to visit (79, 9.6%) and the unavailability of services in the area (117, 14.3%). The complete response for not utilizing mental health services is presented in Fig. 1 below.

### Associations between predisposing and enabling factors and mental health service utilization

After all the predictors were entered into the bivariate analysis model, sex, occupational status, marital status, educational status, monthly income, psychoactive substance use, perceived need, depression, and anxiety symptoms were closely associated with the use of mental health services at *p* $\le 0.25$ and were candidates for the multivariable regression model (Table 3).

**Table 3:** Factors associated with mental health service utilization among study participants in the AM-HDSS, 2022

Variables	COR (95%CI)	AOR (95% CI)	*P* value
Gender			
Male	1.538(1.065, 2.220)	1.099 (.644, 1.876)	.729
Female	-	-	1
Occupation Status			
Employed	1.705(1.157, 2.513)	1.118 (.636, 1.966)	.697
Unemployed			1
Educational Status			
No formal education	-	-	1
Primary school	1.486(.964, 2.289)	1.432 (.842, 2.435)	.186
Secondary education & above	3.270(1.786, 5.988)	3.585 (1.674, 7.674)	.001*
Marital Status			
Single	-	-	1
Divorced	2.147(.550, 8.375)	.766 (.154, 3.818)	.745
Married	2.603(1.404, 4.825)	1.761 (.862, 3.598)	.121
Widowed	.973(.205, 4.613)	.241 (.041, 1.415)	.115
Monthly income			
< 2000	-	-	1
2000–3999	1.933(1.279, 2.921)	2.699 (1.612,4.519)	< .001*
≥ 4000	2.937(1.764, 4.892)	3.030 (1.611, 5.699)	.001*
Lifetime Psychoactive substance			
Yes	2.506(1.755, 3.579)	1.598 (1.027, 2.486)	.038*
No			1
Perceived need for mental health care			
Yes	7.814(4.983, 12.254)	5.894 (3.452, 10.062)	< .001*
No			1
Depression Symptoms			
No depression	-	-	1
Mild—Moderate depression	6.387(4.343, 9.395)	3.313 (2.029, 5.408)	< .001*
Moderate—Severe depression	7.465(3.545, 15.721)	3.092 (1.097, 8.712)	.033*
Anxiety Symptoms			
Minimal Anxiety			1
Mild to moderate Anxiety	6.921(4.752,10.079)	2.813 (1.727, 4.583)	< .001*
Severe Anxiety	6.351(2.060,19.574)	3.499 (.750, 16.317)	.111

^*^Statistically significant variables at *p* $\le 0.05$

The odds of utilizing mental health services were 3.58 times greater for respondents who completed at least secondary level of education (AOR = 3.58, 95% CI: (1.674, 7.67); Respondents with an average monthly income of 2000–3999 birrs (AOR = 2.70, 95% CI: (1.612,4.519) and ≥ 4000 birrs (AOR = 3.03, 95% CI: (1.611, 5.699) were 2.70 and 3.03 times more likely to utilize the service, respectively, than were those who earned less than 2000 birrs. The odds of service utilization were 1.60 times greater for participants who used psychoactive substances than for those who did not (AOR = 1.60, 95% CI: (1.03, 2.49). The odds of utilizing mental health services were 5.89 times greater for participants who had a perceived need for mental health care (AOR = 5.89, 95% CI: (3.452, 10.062).

The odds of utilizing mental health services among participants with a history of mild to moderate depression (AOR = 3.31, 95% CI: (2.03, 5.41) and moderately severe to severe depression (AOR = 3.09, 95% CI: (1.10, 8.71) compared to minimal depression were 3.31 and 3.09 times greater, respectively. Compared to participants with minimal and severe anxiety, those with a history of mild to moderate anxiety were 2.81 times more likely to utilize mental health services (AOR = 2.81, 95% CI: (1.727, 4.583)).

## Discussion

The primary objective of this investigation was to determine the utilization of mental health services among the residents of the Arba Minch Health and Demographic Surveillance Sites System (AM-HDSS) and to identify factors associated with mental health service utilization.

In this study, 15.5% of the participants sought some level of care for their mental health-related concerns. However, the utilization of biomedical or modern mental health care services was again low, with only 15% of those who received care and 2% of the total population receiving care. Only four people from the total sample received care from a trained mental health professional.

Looking at the general utilization will not be informative, as most people do not seek care unless a perceived/actual need for the services arises. However, our study indicates that the utilization of mental health care is still lower among those with these needs. Participants with symptoms of depression and anxiety of varying severity were consistently observed to have low levels of mental health service utilization in our study. Furthermore, only half of the participants who reported needing mental health care sought care.

This study's general utilization of mental health services is comparable to other studies, including those performed in developed countries [6, 21]. However, when comparing the use of modern mental health services by trained professionals, the utilization rate in our study (2%) was significantly lower than that reported in the Czech Republic, United States, Singapore, and Switzerland (17%, 15%, 21.4%, and 20%, respectively) [6, 21, 22, 24].

The observed differences between our study and those in developed countries can be attributed to several factors. Some of these factors include the availability of mental health services in these settings, as well-trained professionals and well-equipped facilities are more likely to be found in developed settings. Literacy and awareness about the cause and treatment of these conditions and other socioeconomic factors, including education and income, are also important factors to consider. Cultural, religious, and spiritual beliefs and practices could also be important determinants, as the causation and treatment of mental health problems are more likely to be biomedical in the developed world, hence leading to better utilization of modern services than in the Ethiopian population, where cultural and spiritual phenomena are often perceived as sources of mental health problems in the literature [17, 19, 26, 35–37].

This significant role of culture and beliefs in the utilization of care and the choice of services is reflected in our study. Although 15.5% utilized some form of mental health service, our study revealed that participants were more likely to use nonmedical mental health care, such as religion and family/friends. The different views about the causation and treatment of mental illnesses in rural settings could explain this. Lack of perceived need, not knowing where to access care, and unavailability of services in the area were discussed by the participants in the study as reasons for not seeking care from modern mental health care providers.

The findings of this study also provide important insight that mental health service utilization patterns differ not only between developed and developed settings, as the utilization of modern mental health services in our study was also lower than that in studies conducted in other low- and middle-income countries. The mental health service utilization in our study was lower than the WHO’s estimation of the utilization of mental health services in low- and middle-income countries (13%) [19]. The utilization rate is also lower than that reported in studies conducted in urban residents in Peru (3.6%) and survivors of genocide in Rwanda (5.3%) [25, 38]. Compared with the primarily rural settings in our study, the urban setting in the Peru study could be a source of potential discrepancy, highlighting the common difference in infrastructure and the socioeconomic divide in urban and rural settings. The possible targeted interventions for individuals living with genocide could contribute to the relatively better utilization of these interventions in the Rwanda study. This highlights that active efforts and policies aimed at targeting, screening, and designing interventions for at-risk and vulnerable individuals/groups of individuals are of public health importance.

The results of our study showed that factors predicting the utilization of mental health services included socioeconomic status, i.e., higher education and higher income, as well as need factors, including perceived need, more significant depressive and anxiety symptoms, and psychoactive substance use. In an unusual association, participants with mild to moderate anxiety were found to be more likely to utilize mental health services than those with minimal and severe anxiety symptoms.

Many other studies have shown education and income to be associated with better utilization of mental health care services [6, 17, 19]. A better understanding of and a means to access these services could be the reason for this positive association, as these variables are often described as predisposing and enabling factors for healthcare utilization. However, the findings of this study contrast with those of a study conducted in Singapore, which reported that higher education and employment were associated with lower help-seeking [24]. The authors of the Singapore study explained the unusual negative association of education and employment with specific cultural issues unique to their community. The authors argue that in their study setting, more educated people often have a more significant perceived burden of conforming to society and fear stigma. In contrast, employed respondents fear the implication of having their conditions known by others on their jobs as reasons for not seeking care. The opposite applies to Ethiopian culture, where less educated individuals fear stigma and social status.

Studies have also shown that having a need to seek care is positively associated with accessing care. These studies include studies conducted in Switzerland, Rwanda, and the United States, which all indicated that need factors, including perceived need, having mental illness or mental health concerns, and substance use, are associated with utilization [6, 21, 38]. As indicated in our study, however, Lund et al. reported that there is still a gap in access to care, even among those who have a perceived need for such services [39].

Overall, our study's findings indicated a significantly lower level of mental health service utilization among those with and without specific actual or perceived needs for services. These findings have significant policy and public health implications for expanding the availability of and access to mental health care services, designing innovative and collaborative solutions to work with traditional and spiritual practitioners and providers, and addressing predisposing factors, including education and improving the socioeconomic status of rural residents.

### Strengths and limitations

This study is the first to study mental health service utilization in southern Ethiopia. The community-based nature of the study is another strength compared to facility-based research, where the sample would be individuals already seeking care. Our study only screened for anxiety and depression as they are the common mental illness in the area. This could limit the generalizability of the findings to other mental health problems.

## Conclusion and recommendation

Our study revealed a significantly low level of utilization of mental health services among the residents of rural southern Ethiopia. These findings indicate a significant treatment gap and suggest the role of poverty and low-level education on individuals’ tendency to seek care. Current efforts to decentralize mental health services, including integrating these services into primary care, should be prioritized as policies to address this growing concern. Collaborations with traditional and religious institutions should also be explored to better serve the people needing these services, as the community tends to rely on them.

## Supplementary Information

Supplementary Material 1.

## Data Availability

The datasets used and/or analyzed during the current study are included in the supplementary materials section of this manuscript.

## Abbreviations

AM-HDSS: Arba Minch Health and Demographic Survey Site
DALYs: Disability Adjusted Life Years
GAD-7: General Anxiety Disorder – 7 items
GBD: Global Burden of Disease
LMIC: Low- and middle-income country
ODK: Open Data Kit
PHQ-9: Patient Health Questionnaire – 9 items
WHO: World Health Organization
